# Can Touch Screen Tablets be Used to Assess Cognitive and Motor Skills in Early Years Primary School Children? A Cross-Cultural Study

**DOI:** 10.3389/fpsyg.2016.01666

**Published:** 2016-10-25

**Authors:** Nicola J. Pitchford, Laura A. Outhwaite

**Affiliations:** Psychology, University of NottinghamNottingham, UK

**Keywords:** assessment, cognitive development, fine motor skills, touch-screens, Malawi, developing countries, cross-cultural comparison

## Abstract

Assessment of cognitive and motor functions is fundamental for developmental and neuropsychological profiling. Assessments are usually conducted on an individual basis, with a trained examiner, using standardized paper and pencil tests, and can take up to an hour or more to complete, depending on the nature of the test. This makes traditional standardized assessments of child development largely unsuitable for use in low-income countries. Touch screen tablets afford the opportunity to assess cognitive functions in groups of participants, with untrained administrators, with precision recording of responses, thus automating the assessment process. In turn, this enables cognitive profiling to be conducted in contexts where access to qualified examiners and standardized assessments are rarely available. As such, touch screen assessments could provide a means of assessing child development in both low- and high-income countries, which would afford cross-cultural comparisons to be made with the same assessment tool. However, before touch screen tablet assessments can be used for cognitive profiling in low-to-high-income countries they need to be shown to provide reliable and valid measures of performance. We report the development of a new touch screen tablet assessment of basic cognitive and motor functions for use with early years primary school children in low- and high-income countries. Measures of spatial intelligence, visual attention, short-term memory, working memory, manual processing speed, and manual coordination are included as well as mathematical knowledge. To investigate if this new touch screen assessment tool can be used for cross-cultural comparisons we administered it to a sample of children (*N* = 283) spanning standards 1–3 in a low-income country, Malawi, and a smaller sample of children (*N* = 70) from first year of formal schooling from a high-income country, the UK. Split-half reliability, test-retest reliability, face validity, convergent construct validity, predictive criterion validity, and concurrent criterion validity were investigated. Results demonstrate “proof of concept” that touch screen tablet technology can provide reliable and valid psychometric measures of performance in the early years, highlighting its potential to be used in cross-cultural comparisons and research.

## Introduction

There are very few cross-cultural tools for assessing early child development. Yet assessment of core cognitive and motor skills in the early years is important for evaluating health and educational interventions, which can help guide policy and best practice to optimize development in early childhood (Sabanathan et al., 2015; Zuilkowski et al., 2016), and enhance the economic potential for disadvantaged children around the world (Heckman, 2006). Here, we consider if touch screen tablet technology can provide an innovative solution to assessing core cognitive and motor skills in the early years that can be used in both low- and high-income countries to identify children at risk of underachievement. We present a new touch screen tablet-based assessment tool that includes measures of core cognitive and motor skills thought to be associated with scholastic progression. We report on initial trials of this new touch screen assessment tool in two representative locations, one high-income country in Europe, the UK, and one low-income country in Sub-Sahara Africa, Malawi, to examine its potential as a cross-cultural tool. These two countries not only differ vastly in gross domestic product, with Malawi being one of the poorest countries and the UK being one of the richest countries in the world (World Bank, 2015^1^), they also differ in culture and education systems. Evaluating the reliability and validity of this new touch screen assessment tool with children attending the early years of primary school from these two countries thus provides a critical test of “proof of concept” that touch screen tablets can be used for cross-cultural psychometric measurements of core cognitive and motor skills.

Sabanathan et al. (2015) highlight five key global developmental domains important in the assessment of a child's developmental progress: (i) cognitive skills, including memory and information processing, (ii) language skills, including receptive and expressive language, (iii) motor skills, including fine motor and gross motor skills, (iv) social and emotional skills; including the ability to understand their own and others emotional states and (v) adaptive behavior skills, including conceptual, social, and practical skills for everyday functioning.

It is thus essential that reliable and valid cross-cultural methods of assessing these key global developmental functions are available across low-to-high-income countries to enable identification of those children most at risk of educational underachievement and in need of intervention support.

While there are a range of cultural specific child development assessment tools (Thompson and Vacha-Haase, 2000), some of which are recommended by funding bodies for global health and education research (Fernald et al., 2009), there are few cross-culturally valid assessments of basic cognitive and motor functioning. There is also limited research evaluating their cross-cultural usability and psychometric properties (Sabanathan et al., 2015). Yet, cross-cultural assessment tools of basic cognitive and motor functions are important if international comparisons of early child development and the theoretical underpinnings are to be sought. However, assessing core developmental skills cross-culturally poses a number of challenges, as outlined in the following section.

### Practical challenges

Most standardized assessments of cognitive, motor, and language skills require strict administration procedures, which necessitates highly trained assessors (Sabanathan et al., 2015), and in some cases controlled laboratory settings (Zuilkowski et al., 2016), which are frequently unavailable in developing countries (Scherzer et al., 2012). Moreover, these assessments are costly and timely to administer. This makes these types of standardized assessments, which are commonly used in high-income countries to identify children at risk of learning difficulties, prohibitive for use in developing countries. To profile strengths and weaknesses of individual children in low-to-middle-income countries, an assessment tool is needed that is low cost, easy to use, and easy to interpret by practitioners with a general training in early child development.

#### Construct bias

Construct bias encompasses cultural differences in how the target skills are operationalized. For example, the construct of intelligence in rural Kenya includes four dimensions: social qualities, practical thinking, comprehension, and academic achievement. Western measures of intelligence correlate with only one aspect of the Kenyan constructs, academic achievement (Grigorenko et al., 2001). An assessment tool that focuses on core cognitive and motor skills would thus alleviate cultural differences in constructs of intelligence, and focus instead on key functions required for scholastic progression.

#### Method and item bias

Methods bias includes differences in assessment administration, such as, the language and medium of delivery and stimuli used, that may favor one group over another (Matafwali and Serpell, 2014). This, in turn, may also impact on item bias, which refers to differences in observed performance despite equal abilities on a particular skill based on participants' cultural or linguistic background. Cross-cultural studies have shown biases in task performance based on different language structures (Jukes and Grigorenko, 2010), and different levels of stimulus familiarity (Callaghan et al., 2012; Zuilkowski et al., 2016). For example, in an attempt to address item bias when adapting a neuropsychological test used in Western cultures to be accessible for the Indonesian population, Prado et al. (2010) changed a picture stimulus of a “bunny” to a “chicken.” However, despite these modifications, young children were still unable to complete the assessment successfully (Abubakar et al., 2008). This may have resulted from other age or language related biases that may have restricted children's participation, in that children might not have understood the task in hand. Assessment tools for cross-cultural use in early child development will benefit from minimal and simple task instructions, that require non-verbal responses to be made, and use stimuli that are acquired early in life and transcend cultures, such as basic shapes and colors (Bornstein et al., 1976).

#### Lack of normative data

Standardized assessments that are commonly used in high-income countries often lack normative data for low-to-middle-income countries, rendering them unsuitable for use in the developing world. Standardizing assessments is non-trivial and traditional approaches that make use of paper and pencil administration require high investment in time and resources to produce reliable norms that span the developmental timeframe when key cognitive, motor, language and scholastic skills are learnt (preschool to late adolescence). In developing an assessment tool to be used in low-to-middle-income countries innovative methods of collecting normative data that are reliable and rapid are needed and touch screen assessment tools need to be sensitive to maturational processes.

### Recent progress

In spite of these challenges, recent progress has been made in developing valid cognitive and motor assessments for use in specific developing countries, particularly in the fields of health and education (Jukes and Grigorenko, 2010). For example, cognitive assessments, including motor skills, executive function, and language abilities, have been developed specifically for Zambia (Serpell, 1974; Ezeilo, 1978; Fink et al., 2013), rural Kenya (Kitsao-Wekulo et al., 2012), Bangladesh (Khan et al., 2013), and Malawi (Gladstone et al., 2009, 2010) populations. Assessment designed to determine young children's developmental milestones have also been developed in South Africa (Boyede et al., 2016), Malawi (Gladstone et al., 2009, 2010), Kenya (Prado et al., 2010), Nigeria (Eseigbe, 2013), and Cambodia (Ngoun et al., 2012).

These assessments are designed to be administered by trained assessors and usually involve observational checklists (e.g., Gladstone et al., 2009, 2010; Boyede et al., 2016), parental reports (e.g., Ngoun et al., 2012), or require a battery of specific resources (e.g., Jukes and Grigorenko, 2010). These methods, while insightful, can be expensive and timely to administer, and usually focus on measuring developmental milestones that typify early child development prior to school entry (Gladstone et al., 2009, 2010). Thus, they may not be sustainable for use outside of the research context. They are also country-specific so cannot be used to make cross-cultural comparisons. A generic assessment tool is thus needed, that is both reliable and valid across different cultures, which is cross-validated with scholastic performance, to enable cross-cultural studies of child development to be conducted, and a universal framework of factors that influence progress through school to be developed.

Several international bodies, including the Malawi Institute of Education (2014), have called for modern forms of data collection that utilize mobile devices, which have the ability to collect valid and reliable outcome data, and reduce time and monetary costs. Tablet-based versions of international numeracy and literacy assessments, such as, the Early Grade Mathematics Assessment, EGMA (Brombacher, 2010) and the Early Grade Reading Assessment, EGRA (Gove and Wetterberg, 2011) have been developed by RTI international. However, these require a trained evaluator to administer questions and record individual children's responses through the tablet. They do not capitalize on the touch screen tablet technology that can be used to record responses directly from individual children in response to particular tasks. As such, the tablet versions of EGMA and EGRA still require one-to-one administration, which is costly both in time and human resources.

### Current study

We have developed a new touch screen tablet-based assessment tool for cross-cultural comparisons of core cognitive and motor skills in primary school children that addresses the challenges and limitations discussed. The new assessment tool was designed by the first author and programmed by onebillion, a UK-based charity. We report on the initial stage of its development, through trials with children attending the first 3 years of primary school in Malawi and the first year of primary school in the UK. To demonstrate “proof of concept” we need to show that the touch screen assessment tool is reliable and valid across cultures.

#### Constructs measured

This new touch screen assessment tool includes measures of manual processing speed, manual coordination, short-term memory, visual attention, working memory, and spatial intelligence. These cognitive and motor measures were chosen because of their close association with the development of fundamental scholastic skills, such as mathematics and literacy (e.g., Nunes et al., 2007; Berg, 2008; Mulder et al., 2010; Westerndorp et al., 2011; Bourke et al., 2014; Simms et al., 2014; Pitchford et al., 2016). Accordingly, a measure of scholastic skill - mathematics - that is taught from the start of formal schooling in both Malawi and the UK was also included to cross-validate the new assessment tool.

#### Item stimuli

The stimuli used to assess core cognitive and motor skills centered on basic shapes and colors, as these are easily discriminable, acquired at an early age, and commonly occur in urban and semi-urban environments (e.g., Bornstein et al., 1976). Basic shapes, such as squares, rectangles and circles, are represented even in rural environments in developing countries, such as village houses and churches, and basic colors are frequent in the clothing worn by both rural and urban people.

#### Assessment delivery

This new assessment tool utilizes touch screen technology as its method of delivery and recording responses from individual child. All tasks required a non-verbal, manual, response, to be made. Recent research with high-income countries has demonstrated the usability, affordance and potential for using tablet technology for collecting cognitive development data with young children in a research setting (Semmelmann et al., 2016). However, to our knowledge, our touch screen assessment tool is the only direct measure of child performance across a range of neuropsychological tasks shown to be associated with developmental disorders and scholastic progression that has been trialed across both low- and high-income country contexts.

The use of touch screen tablet technology in the assessment of cognitive and fine motor abilities offers several unique affordances. Tablet technology is lightweight and eliminates the need for other devices that may rely on developed motor skills (Donker and Reitsma, 2007; Kucirkova, 2014). Even young children (aged 2–3 years) have the required motor skills to use touch screen technology (Nacher et al., 2015). Furthermore, apps are available for assessing and training fine motor skills that are grounded in occupational therapy techniques (e.g., Dexteria, Kizony et al., 2016; Short et al., in press) thus illustrating that touch screen technologies are suitable for use in assessing core skills in primary school aged children. Furthermore, tablet technology allows standardized procedures for all children and so eliminates researcher or teacher bias and reduces measurement error. Consequently, there has been an increase in the use of touch screen technology in cognitive assessments in the West. For example, Pearson Education Ltd^2^ have developed Q-interactive, a tablet-based tool for administering a number of cognitive assessments traditionally administered in a paper and pen format. Despite the advances in tablet technology based assessments in high-income countries, there is a significant gap in resources for developing countries that needs to be addressed.

## Methods

We evaluated this new touch screen tablet-based assessment tool for reliability and validity in early years populations from both Malawi and the UK. Reliability and validity measures were based on the basic psychometric properties used to evaluate child development assessment tools outlined by Sabanathan et al. (2015).

### Participants

The Malawi sample consisted of 283 children from Standards 1–3 (the first 3 years of education in Malawi) attending a state primary school located in an urban area of Lilongwe, the capital of Malawi. The sample consisted of 144 males and 139 females. Age ranged between 73 and 161 months^3^ (*M* = 97.15 months, *SD* = 15.16 months; median age = 94.00 months). Any learning difficulties were unknown. The Ministry of Education in Malawi gave consent for the study to take place and selected the participating primary school. Consent was also obtained from the parent association at the primary school and the Community Chief of the region where the primary school is located.

The UK sample consisted of 70 pupils in Foundation 2 (the first year of compulsory education in the UK) attending a primary school situated in Nottingham, a metropolitan city in the United Kingdom. The sample consisted of 39 males and 31 females. Age ranged between 50 and 69 months (*M* = 60.81, *SD* = 4.98). Two children in the sample were identified to have special educational needs in the form of mild autistic spectrum disorder. Eight children were absent at the time of data collection for standardized measures used for validation purposes and so were excluded from the associated data analyses. This study was granted ethical approval from the ethics committee at the School of Psychology, University of Nottingham, and written parental consent was obtained for all participating children prior to study commencement.

For each sample, outliers (defined as 2 standard deviations or more above and below the group mean) for each of the cognitive and motor tasks included in the new assessment tool were excluded from the analysis. Table 1 describes the final Malawi and UK samples for each task.

**Table 1:** **Structure of the Malawi and UK samples for each task**.

**Task**	**Malawi**					**UK**				
	***n***	**Gender**	**Age (months)**			***n***	**Gender**	**Age (months)**		
		**M:F**	**Range**	**M (SD)**	**Median**		**M:F**	**Range**	**M (SD)**	**Median**
Manual processing speed	261	134:127	73–161	97.54 (15.30)	96.00	62	36:26	50–69	60.94 (4.82)	61.00
Manual coordination	218	107:111	74–161	100.82 (14.75)	99.00	64	36:28	50–69	60.94 (4.75)	61.00
Short-term memory	215	105:110	74–161	99.30 (14.27)	98.00	69	38:31	50–69	60.72 (4.96)	61.00
Visual attention	233	117:116	73–161	98.70 (15.50)	98.00	62	36:26	50–69	60.51 (5.01)	61.00
Working memory	221	109:112	74–161	99.29 (14.50)	98.00	67	36:31	50–69	60.96 (4.88)	61.00
Spatial intelligence	223	105:118	74–161	99.35 (14.75)	98.00	66	36:30	50–69	60.74 (5.07)	61.00
Mathematics	266	132:134	73–161	96.42 (15.06)	94.00	59	36:23	50–69	60.37 (5.09)	60.00

### Tablet-based assessment measures

**Figure 1:**
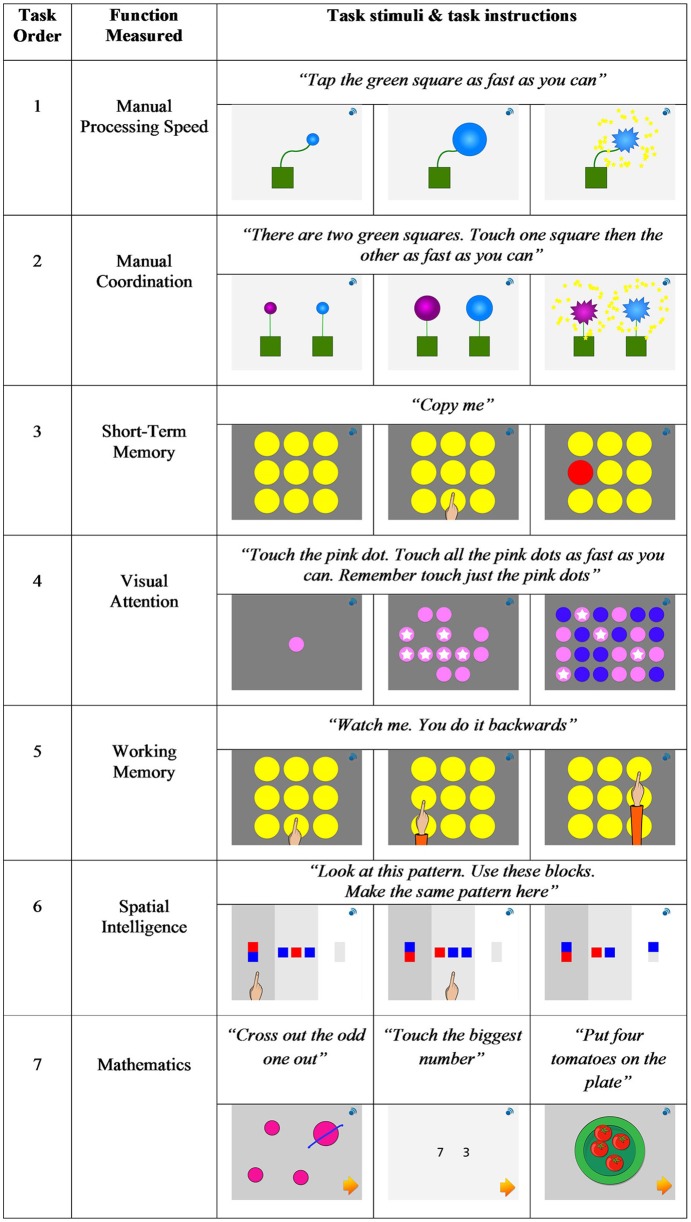
**Schematic illustration of tasks included in the new touch screen tablet-based assessment of cognitive and mathematical skills for primary school children**.

All participating children were assessed on six measures of cognitive development: manual processing speed, manual coordination, short-term memory, visual attention, working memory, and spatial intelligence. A measure of mathematics was also given, that included assessment of both curriculum and conceptual knowledge. Each task is described in the following section and is illustrated in Figure 1.

#### Manual processing speed

A single-finger-tapping task was used to assess manual processing speed (see Witt et al., 2008). Using the index finger of their dominant hand children were required to tap a green box displayed on the screen continually, as fast as they could, which caused a blue balloon to increase in size. The task was complete when the child had tapped the green box 30 times causing the balloon to pop. An overall measure of manual processing speed was calculated from the mean completion time across the two trials.

#### Manual coordination

Manual coordination was assessed using an alternating finger tapping task (see Witt et al., 2008). Similar to the manual processing speed task, stimuli consisted of two green boxes and one blue and one purple balloon. Children were required to tap each of the two green boxes alternatively, with the index finger of their left and right hand, to pop the two balloons. Balloons would only increase in size if the child tapped each green box alternately with their left then right index finger. Each box required tapping 30 times in sequence for the balloon to pop. An overall measure of manual coordination was calculated from the mean completion time across the two trials.

#### Short-term memory

A forward spatial span task was used to assess short-term memory, similar to that used by Brunetti et al. (2014). Children were presented with a three-by-three grid of yellow circles. The virtual instructor demonstrated the pattern to be recorded by the child by touching the yellow circles. When the demonstrator touched a yellow circle it turned red, momentarily, until the demonstrator touch the next circle in the sequence. Children were then required to repeat the order they had been presented. The number of circles included in the pattern increased in line with progression through the test, starting at 1 and increasing to 9. The task discontinued after three successive incorrect trials. The number of trials completed correctly gave the overall short-term memory score.

#### Visual attention

Visual attention was assessed through a speeded search task, similar to that used by Pitchford et al. (2011). Before each of three experimental trials, children were presented with a baseline practice trial in which they were shown a single colored dot, followed by an array of either 8, 12, or 16 same colored dots, which they were instructed to touch as fast as they could. In the experimental trials, children were required to distinguish and touch all the colored dots given in the practice trial from a display of different colored distracter dots. For each trial, time taken to complete the baseline trial was subtracted from the time taken to complete the experimental trial, thus generating a measure of visual attention that was not confounded by manual processing speed. An overall measure of visual attention was derived from the mean response times taken to complete the three experimental trials.

#### Working memory

A backward spatial span task was used to assess working memory, similar to that used by Brunetti et al. (2014). The task followed the same layout and characteristics as the short-term memory, forward spatial span, task, except that children were required to repeat the presented pattern backwards. The number of circles included in the pattern increased in line with progression from 2 to 9. The task discontinued after three successive incorrect trials. An overall working memory score was calculated on the number of correct trials completed.

#### Spatial intelligence

Spatial intelligence was assessed using a two-dimensional pattern-processing task, similar to the three-dimensional Block Design task used in standardized assessments (e.g., Wechsler, 2003). The task required children to reconstruct a two-dimensional pattern using simultaneously displayed pattern squares. The number of pattern squares available depended on the size of the presented pattern that children were required to recreate. The task discontinued after three successive incorrect trials. An overall spatial intelligence score was obtained from the number of correct patterns recreated.

#### Mathematics

A test consisting of 98 items, measuring different aspects of curriculum and conceptual knowledge was used to assess mathematics. The curriculum questions were based on the content of the onebillion mathematics apps (Pitchford, 2015) that are grounded in the UK national curriculum, and cover topics such as counting, addition, subtraction, and shape and space recognition. The mathematics curriculum in Malawi is based on the UK curriculum and places a strong focus on the acquisition of numeracy skills (Chirwa and Naidoo, 2014). The conceptual questions were based on the Early Grade Mathematics Assessment (EGMA; Brombacher, 2010) and the Numerical Operations subtest of the WIAT-II (Wechsler, 2005; see Pitchford, 2015). Concepts assessed included symbolic understanding, numbers in relation to each other, number line understanding, counting, number sense (quantity estimation), simple and complex addition and subtraction, multiplication, and division. Task difficulty increased in line with task progression and discontinued after three successive incorrect answers. An overall mathematics score was determined from the total number of questions answered correctly.

### Standardized measures (UK only)

To assess the criterion validity of the new touch screen tablet assessment tool, children in the UK sample were also given two standardized measures of cognitive development, namely the Block Design and Symbol Search subtests from the WPPSI-III (Wechsler, 2003). These Western based standardized measures were chosen as they are similar to the cognitive skills measured in the new tablet assessment. In particular, we predicted that performance on the Block Design subtest from the WPPSI-III should correlate with the task of spatial intelligence on the new touch screen tablet assessment as both are designed to measure spatial reasoning skills. Likewise, we expected performance on the Symbol Search subtest from the WPPSI-III to correlate with the tasks of manual processing speed, short-term memory, and working memory. This is because the Symbol Search subtest from the WPSSI-III is a measure of cognitive processing speed which is known to be dynamically related to working memory (Kail and Salthouse, 1994; Fry and Hale, 1996) and in young children working memory and short-term memory are highly correlated (Hornung et al., 2011; Aben et al., 2012).

#### Block design

The Block Design subtest of the WPPSI-III requires children to recreate block patterns presented as a constructed model or picture using one or two colored blocks within a specified time. The task is designed to test ability to analyse and synthesize abstract visual stimuli and is an assessment of non-verbal, spatial intelligence, and visual-motor coordination (Sattler, 2001). This measure has good internal consistency for children aged 4–5 years, ranging from 0.76 to 0.85, as reported in the test manual (Wechsler, 2003, p. 52). Raw scores were used.

#### Symbol search

The Symbol Search subtest of the WPPSI-III requires children to identify whether or not an abstract target symbol is present amongst an array of other similar symbols. The task is designed to assess processing speed and incorporates visual short-term memory and visual-motor coordination (Sattler, 2001). Similar to the Block Design, this measure has good internal consistency for children aged 4–5 years, ranging from 0.76 to 0.85, as reported in the test manual (Wechsler, 2003, p. 52). Raw scores were used.

### Procedure

All children completed the touch screen tablet assessments independently, which were delivered through an individual iPad mini connected to a set of headphones, whilst they were sat on the floor of their classroom. Tasks were presented in the order outlined above and as listed in Figure 1. A virtual instructor delivered task instructions in the child's local language (Chichewa in Malawi; English in the UK). The child could repeat task instructions on demand by touching a small button in the corner of the screen. The virtual instructor demonstrated this at the start of the assessment tool, during a familiarization task.

The familiarization task included at the start of the new tablet assessment tool taught children how to perform the operations required for the using the tablet to complete the individual tasks. For example, demonstrations were given by the virtual instructor in how to select and move objects around the touch screen then children were given the opportunity to practice these actions. The familiarization task also had immediate positive feedback on correct responses in the form of a tick and high-pitched sound. Feedback was only given during the familiarization task to encourage children who were using the tablet for the first time.

**Figure 2:**
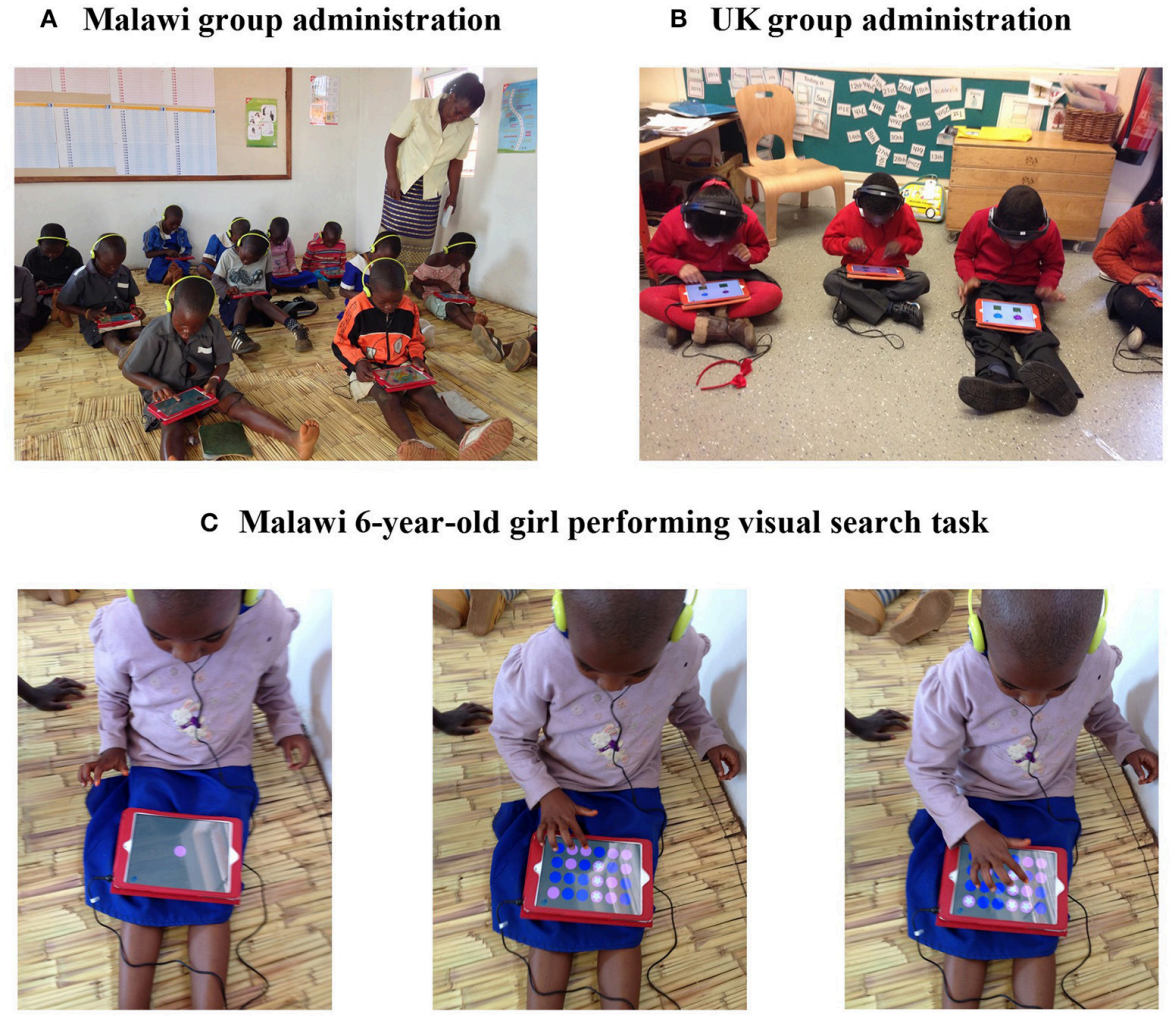
**Group administration of the new touch screen tablet-based assessment with primary school children in Malawi (A) and the UK (B)**. Six-year-old girl in Malawi performing the visual attention task **(C)**.

In Malawi, the new tablet assessment tool was administered in groups of up to 50 children. The total group of 283 children completed the new assessment tool on two occasions, with an interval of 8-weeks between administrations. In the UK, the new tablet assessment tool was administered in groups of up to 15 children. The total group of 70 children completed the new assessment tool just once. Figure 2 illustrates the assessment tool being administered to groups of children in Malawi and the UK.

For both samples, individual tasks were demonstrated to children by the researcher before the start of each task. In Malawi, teaching staff and a volunteer from the Voluntary Service Overseas supervised the group administration, so as to provide language support for the English-speaking researcher (first author) whilst she demonstrated the tasks. Data for individual children was recorded by the tablets and later retrieved through an Internet server hosted by onebillion, the UK charity supporting this project. For the UK sample, after completing the new tablet assessment tool with groups of children, the two standardized measures were given in a separate session to individual children, by the researcher (second author), in a quiet area, free from distraction, in their familiar school environment. Block Design was given first, followed by Symbol Search.

## Results

To evaluate different aspects of reliability and validity of the new touch screen tablet-based assessment tool a series of correlations was conducted for each sample. A two-tailed level of probability was adopted in all analyses, despite some directional hypotheses being made.

Tables 2, 3 report Cronbach's Alpha and Pearson's Product Moment correlation coefficients for each of the following investigations.

**Table 2:** **Reliability and validity analyses for Malawi and UK samples**.

**Task**	**Correlations (*r*)**								
	**Malawi**				**UK**				
	**Split-half reliability**	**Test-retest reliability**	**Age**	**Predictive criterion validity^a^**	**Split-half reliability**	**Age**	**Predictive criterion validity^a^**	**Criterion validity**	
								**Block design**	**Symbol search**
Manual processing speed	0.50^**^	0.35^**^	−0.29^**^	−0.23^**^	0.53^**^	−0.35^**^	−0.18	0.03	−0.25^*^
Manual coordination	0.93^**^	0.16^**^	−0.16^*^	−0.04	0.88^**^	−0.10	0.03	0.05	0.07
Short-term memory	–	0.34^**^	0.13	0.21^**^	–	0.10	0.23^*^	0.17	0.37^**^
Visual attention	0.40^**^	0.42^**^	−0.34^**^	−0.34^**^	0.44^**^	−0.25^*^	−0.16	−0.11	−0.16
Working memory	–	0.05	0.07	−0.06	–	0.04	0.29^*^	0.24^*^	0.36^**^
Spatial intelligence	–	0.12	0.13	0.20^**^	–	0.08	0.31^**^	0.33^*^	0.15
Mathematics	–	0.73^**^	0.39^**^	–	–	0.30^*^	–	–	–
		(*n* = 77)							

** *p < 0.001*,

* *p < 0.05*.

a *Predictive criterion validity: correlation coefficients for each of core cognitive and motor tasks and Mathematics. A reduced sample size of 77 pupils was used for the Malawi test-retest reliability of the Mathematics task*.

**Table 3:** **Convergent construct validity: correlation matrix across all six tasks for Malawi and UK samples**.

**Task**	**Correlations (*r*)**									
	**Malawi**					**UK**				
	**Manual processing speed**	**Manual coordination**	**Short-term memory**	**Visual attention**	**Working memory**	**Manual processing speed**	**Manual coordination**	**Short-term memory**	**Visual attention**	**Working memory**
Manual coordination	0.17^**^	–				0.16	–			
Short-term memory	−0.9	−0.08	–			−0.20	−0.16	–		
Visual attention	0.26^**^	0.13^*^	−0.18^**^	–		0.16	0.14	−0.18	–	
Working memory	−0.07	0.13^*^	0.003	0.03	–	−0.11	−0.17	0.31^**^	−0.02	–
Spatial intelligence	−0.04	−0.01	0.14^*^	−0.24^**^	0.05	0.01	−0.15	0.48^**^	−0.18	0.31^**^

** *p < 0.001*,

* *p < 0.05*.

### Split-half reliability

The three timed tasks (manual processing speed, manual coordination, and visual attention) included more than one trial so internal consistency was investigated for each of these tasks, for each sample, by correlating performance across trials using Cronbach's Alpha correlation coefficients. For both samples, significant, moderate to strong, positive correlations were found across trials for each of the three timed tasks (Table 2).

### Test-retest reliability

The Malawi sample was given the new touch screen assessment tool on two occasions, separated by a 8-week interval, enabling consistency over time to be investigated each task, by correlating performance across the first and second administration^4^. Significant, moderate to strong, positive correlations were found across repeated administration of all tasks, except for working memory and spatial intelligence where weak correlations were found (Table 2).

### Face validity

The new touch screen assessment tool should be sensitive to developmental progression, in that performance should increase with age. Thus, face validity was established by correlating age (months) with task performance for all of the measures included in the new assessment tool. Results revealed negative, moderate correlations with age for each of the three timed tasks (manual processing speed, manual coordination, and visual attention), demonstrating faster performance by older children. For both samples, these age-related correlations were significant, except for manual coordination in the UK sample where a weak, non-significant, correlation was found. Likewise, positive correlations were found with age for each of the three core accuracy tasks (short-term memory, working memory, and spatial intelligence), demonstrating better performance by older children. However, in both samples, only weak correlations were found with the three tablet-based tasks measuring accuracy of response, which were not significant, suggesting these measures are not particularly sensitive to developmental progression. In contrast, age correlated significantly with mathematics, as moderate and positive correlations were found of similar strength across cultures, illustrating that with increasing age knowledge of mathematical curriculum and concepts increases, as expected over the first years of primary school (Table 2).

### Convergent construct validity

The three tasks measuring speed of response (i.e., manual processing speed, manual coordination, and visual attention) should correlate positively with one another across both samples. Likewise, the three tasks measuring performance accuracy (i.e., short-term memory, working memory, and spatial intelligence) should correlate positively with one another across both samples. To investigate convergent construct validity for the three tasks involving speed of response and the three tasks measuring accuracy of response a correlation matrix was produced, with partial correlations controlling for age.

As predicted, in both samples, positive correlations, of similar strength, were found amongst the three tasks measuring speed of response. These were significant for the larger Malawi sample but were not significant in the smaller UK sample (see Table 3). Likewise, in both samples, positive correlations were found amongst the three tasks measuring performance accuracy. Whilst moderate, significant, correlations were found in the UK sample amongst all three accuracy tasks, in the Malawi sample only the correlation between short-term memory and spatial intelligence was significant. Both correlations involving working memory were weak and not significant in the Malawi sample, suggesting the working memory measure within this sample has limited construct validity (see Table 3).

### Predictive criterion validity

To further explore how the six tasks included in the new assessment tool predicted mathematical knowledge, partial correlations were performed for each tablet-based task and mathematics, controlling for age. In addition, to establish the contribution that each of the core cognitive and motor tasks made to mathematics performance, stepwise linear regression was used by entering the three accuracy tasks at step 1 followed by the three speeded tasks at step 2.

Results showed the core cognitive and motor tasks included in the new touch screen assessment tool correlated with mathematics performance in the expected direction. As shown in Table 2, negative correlations were found between each of the three timed tasks (manual processing speed, manual coordination, and visual attention) and mathematics for both samples, and these were of moderate strength and significant in the Malawi sample, except for manual coordination. Although in the predicted direction, weak correlations were found in the UK sample between each of the three timed tasks and mathematics, none of which were significant. For the three accuracy measures (short-term memory, working memory, and spatial intelligence) and mathematics, significant, positive correlations, of moderate strength, were found in both samples, except for working memory in the Malawi sample where a very weak negative correlation was found.

Stepwise linear regression analyses revealed a similar amount of variance in mathematics performance was accounted for by the core cognitive and motor tasks included in the new touch screen assessment tool. As shown in Table 4, for both samples, 15% of the total variance was accounted for by the tablet-based cognitive and motor tasks. Whilst the model fits were significant for the larger Malawi sample, the model fits were not significant for the smaller UK sample, indicating a lack of power in the UK sample with six predictor variables. For the Malawi sample, the tasks of spatial intelligence and manual processing speed contributed significantly to the model fit, accounting for 8 and 7% of the total variance respectively. In the UK sample, the only significant predictor of mathematical performance was manual processing speed, which accounted for 10% of the total variance.

**Table 4:** **Predictive criterion validity: linear regression models to examine variance in mathematics accounted for by accuracy and timed tasks in Malawi and UK samples**.

**Model**	**Variable(s)**	**Model**		**Significance**	**Change**		**Unstandardized coefficients**	**Standardized coefficients**	**Significance**
		***R***	***R*^2^**	***F* (*df*), *p***	**Δ*R*^2^**	**Sig. Δ*F***	***B*, Std. Error**	**Beta**	***t, p***
**MALAWI**									
1	**Accuracy tasks**	0.28	0.08	3.37 (3, 123),	0.08	***p*** = **0.021**			
	Short-term memory			***p*** = **0.021**			1.13, 2.78	0.16	1.82, *p* = 0.071
	Working memory						−1.49, 1.34	−0.10	−1.11, *p* = 0.269
	Spatial intelligence						2.03, 0.90	0.20	**2.25,** ***p*** = **0.027**
2	**Accuracy tasks**	0.39	0.15	3.51 (6, 120),	0.07	***p*** = **0.019**			
	Short-term memory			***p*** = **0.003**			0.95, 0.61	0.13	1.55, *p* = 0.123
	Working memory						−1.54, 1.31	−0.10	−1.18, *p* = 0.241
	Spatial intelligence						1.74, 0.89	0.17	1.95, *p* = 0.053
	**Timed tasks**								
	Manual processing speed						−0.001, < 0.0001	−0.20	−**2.29,** ***p*** = **0.024**
	Manual coordination						0.00002, < 0.0001	0.02	−0.24, *p* = 0.813
	Visual attention						−0.007, 0.005	−0.14	−1.55, 0.123
**UK**									
1	**Accuracy tasks**	0.23	0.05	0.74 (3, 40),	0.05	*p* = 0.534			
	Short-term memory			*p* = 0.534			−0.37, 1.12	−0.06	−0.33, *p* = 0.743
	Working memory						0.66, 2.41	0.05	0.28, *p* = 0.785
	Spatial intelligence						1.29, 0.98	0.23	1.32, *p* = 0.194
2	**Accuracy tasks**	0.39	0.15	1.13 (6, 37),	0.10	*p* = 0.232			
	Short-term memory			*p* = 0.363			−0.74, 1.12	−0.11	−0.66, *p* = 0.514
	Working memory						−0.14, 2.45	−0.01	−0.06, *p* = 0.956
	Spatial intelligence						1.85, 1.00	0.33	1.84, *p* = 0.073
	**Timed task**								
	Manual processing speed						−0.003, 0.001	−0.33	−**2.06,** ***p*** = **0.047**
	Manual coordination						0.00007, < 0.0001	−0.05	−0.29, *p* = 0.774
	Visual attention						0.01, 0.01	0.11	0.64, *p* = 0.524

*Significant results highlighted in bold*.

### Concurrent criterion validity

The UK sample was also given two standardized subtests of the WPSSI-III. This enabled concurrent criterion validity to be investigated by conducting partial correlations between the six core cognitive and motor tasks included in the new touch screen assessment tool and performance on the two standardized sub-tests of the WPSSI-III, using raw scores and controlling for age.

As predicted, a significant, positive correlation, of moderate strength, was found between Block Design and the tablet measure of spatial intelligence, as both tasks were designed to measure spatial reasoning skills (see Table 2). In addition, the tablet measure of working memory also correlated significantly with Block Design, presumably because it was a visuo-spatial working memory task. Likewise, significant correlations, of moderate strength, were found in the predicted direction between Symbol Search and the tablet measures of manual processing speed, short-term memory, and working memory. This was expected because the Symbol Search subtest of the WPSSI-III is designed to measure cognitive processing speed, which is dynamically related to working memory and short-term memory in early childhood.

## Discussion

We have demonstrated “proof of concept” that touch screen tablet technology can be used for cross-cultural assessments of core cognitive and motor functions associated with scholastic progression, in the early primary years. The new assessment tool that we describe was trialed with samples of children attending the first years of primary school in two countries, one high-income (UK) and one low-income (Malawi), which differ radically in culture and educational context. Despite these differences, results showed remarkably similar patterns of reliability and validity across samples, for children's performance on the new touch screen assessment tool, demonstrating its potential to be used in cross-cultural comparisons and research.

Results showed the new touch screen assessment tool had good internal consistency for timed measures including multiple trials in both the Malawi and UK samples. In the Malawi sample, moderate test-retest reliability was shown for the majority of tasks, and for both samples, reasonable face validity was demonstrated, in that task performance correlated with age. Specifically, age correlated negatively with performance on the three tasks measuring speed of response and positively (albeit weakly) with the three tasks where accuracy of response was measured. These results are consistent with previous research demonstrating reduced reaction times on a computer-assisted reaction time task and increased performance on Ravens progressive matrices in line with chronological and educational age (Van de Vijver and Brouwers, 2009). In addition, significant, positive correlations with age and mathematics were found across cultures, demonstrating that touch screen technology can provide a valid means of measuring scholastics skills that are taught from the start of primary school.

Reasonable convergent construct validity was also shown across cultures for the six tasks included in the new touch screen assessment tool. As predicted, in both samples, the three tasks measuring speed of response correlated with one another, as did the three tasks measuring accuracy of response. This corroborates a robust body of evidence demonstrating interrelations between different cognitive and motor skills during development (see Diamond, 2000, 2007, for reviews). However, within the Malawi sample, both correlations involving working memory were weak and not significant. The lack of correlation with working memory within the Malawi sample might arise from generally low levels of performance on this task. Despite a broader age range (first 3 years of primary school) in the Malawi sample than the UK sample, performance on the working memory task was significantly lower in the Malawi sample than the younger sample of UK children [Malawi, *M* = 0.48, *SD* = 0.71; UK, *M* = 0.79, *SD* = 0.94, *t*_(286)_ = 2.87, *p* = 0.004].

Working memory typically starts to develop around 4 years in Western cultures (Gathercole et al., 2004). This coincides with when children typically start school in the UK and formal schooling enhances working memory (Kosmidis et al., 2011). However, in Malawi, formal schooling and quality education is limited, due to high student-teacher ratios, a shortage of qualified teachers, short school days, and limited teaching resources (Hubber et al., 2016). Consequently, Malawi education relies on rehearsal, rather than deeper forms of learning involving simultaneous storage and processing, which typify UK classroom activities. Thus, the education context in Malawi may account for the observed poor working memory performance and lack of correlations between working memory and the other cognitive tasks measuring performance accuracy found here.

Similarly, predictive criterion validity was established across cultures. When the three tasks measuring accuracy of response and the three tasks measuring speed of response were entered into a regression model predicting mathematical ability, 15% of the total variance was accounted for within each sample. Manual processing speed contributed uniquely to the model fits in both samples, indicating this measure is a cross-cultural predictor of early mathematical ability. Recent studies have identified fine motor skills to be a significant predictor of mathematical ability in Western populations (Becker et al., 2014; Cameron et al., 2016; Pitchford et al., 2016). However, the tasks used to measure fine motor skills in these studies often include an aspect of spatial processing (Barnhardt et al., 2005; Simms et al., 2016), making it difficult to determine if it is the spatial or fine motor skills that are predictive of early mathematical ability. Whilst the two tasks of fine motor skill included here have limited spatial processing, only the task of manual processing speed predicted mathematical ability. This indicates that it is the measurement of processing speed, rather than fine manual control *per se*, that is contributing significantly to predicting early mathematical ability, especially considering all of the touch screen tasks involved a motoric response. This corroborates previous research that has shown verbal processing speed measures to be predictive of mathematical ability in preterm populations (Mulder et al., 2010), and suggests that processing speed might be a domain general predictor of early mathematical ability across cultures.

Finally, for the UK sample, good concurrent criterion validity was shown. As predicted, the new touch screen assessments of spatial intelligence and working memory correlated significantly with Block Design from the WPPSI-III. Likewise, the new touch screen assessments of manual processing speed, short-term memory and working memory correlated with Symbol Search from WPPSI-III.

Overall, these results demonstrate a valuable first step in the development of a cross-cultural touch screen assessment tool for measuring core cognitive and motor skills in primary school children. However, it is important to acknowledge that many of the correlations reported here are weak to moderate in strength, indicating that whilst initial “proof of concept” has been demonstrated, further refinement of the tasks included in this new touch screen assessment tool is needed. Despite these limitations, we have shown that using tablet technology with simple tasks that employ basic stimuli can address several of the challenges that arise in cross-cultural comparisons of child development. For example, with the new touch screen assessment tool, children are exposed to the exactly the same standardized procedures and protocols, and task instructions are given in the child's first language, thus eliminating bias induced through different assessors (Sabanathan et al., 2015), and the need for trained assessors, which in low-income countries are in very short supply. This means that the new touch screen assessment tool is easy to implement by educational staff with limited experience of standardized assessments. We have also shown that group administration is possible with tablet technology, thus reducing the time and human resources required for one-to-one administration of more traditional standardized tasks. In turn, assessments using touch screen tablet technology could become valuable and efficient tools for evaluating core cognitive and fine motor skills in primary school children that can be administered quickly, to groups of children, by class teachers.

Assessment delivery in the child's first language also addresses potential methods and item bias. The importance of the child's first language in assessments and education is widely emphasized (e.g., GEM Report, 2016) as less variance arises in academic performance if children are assessed in their first, rather than second, language (Pretorius and Mampuru, 2007). The new touch screen assessment tool can be readily adapted to other languages, as the task instructions are simple, the task stimuli comprise of basic shapes, and a virtual instructor demonstrates tasks to the child. These features make the new touch screen assessment tool suitable for use in different educational contexts and cultures.

Interestingly, potential differences in exposure to touch screen tablet technology across children in the two countries where the new assessment tool was piloted did not appear to effect performance. Whilst 70% of UK children have access to touch screen technology at home (Ofcom, 2014^5^) and in school (Clarke, 2014) most children in Malawi have limited exposure to touch screen technology. Thus, the Malawi sample had reduced familiarity with the medium of delivery compared to the younger UK sample. Steps were taken to address this potential exposure bias through the inclusion of a pre-assessment task that aimed to familiarize children with the required drag and drop and tapping movements needed to complete the cognitive, motor and mathematics tasks. Our results showed similar patterns of performance across countries for the tasks included in the new tablet-based assessment, except for working memory, demonstrating that this technology can be used effectively to assess core cognitive and fine motor skills even in children with limited exposure to using touch screens.

Also, using stimuli that are simple geometric shapes rather than pictorial representations addresses problems highlighted in previous research that were limited by using culturally bound pictorial stimuli (e.g., Prado et al., 2010). Although it could be argued that geometric shapes are more familiar in high-income countries compared to the developing world (Roberson et al., 2002), basic geometric shapes and colors are represented in low-income countries, in both urban and rural environments. Mathematics is also one of the key “Learning Areas” in the national curriculum delivered in most low-income countries, such as Malawi (Chirwa and Naidoo, 2014), indicating that primary school children in low-income countries should have some experience of geometric shapes. The similar patterns of performance shown in our study across children from Malawi and the UK demonstrates the appropriateness of using basic shapes and colors for task stimuli in cross-cultural assessments of cognitive and motor skill.

For this new tablet-based assessment tool to be used to effectively to target individuals at risk of learning difficulties and in need of intervention, further development is needed in three key areas. Firstly, the current tasks require refinement to ensure the sensitivity of this new assessment tool to different ages and cultures. Results from the current study show age correlated with most of the tasks included in the new touch screen assessment tool, despite differences in the age range of the UK and Malawi samples. When the new touch screen assessment tool is highly sensitive to a broader range of ages than investigated here, this will enable the effects of maturation from schooling to be disentangled, as the age at which children start formal schooling differs across cultures. Criterion validity also needs to be evaluated in low-income countries. This may prove difficult, however, as, in many low-income countries there is no “gold standard” assessment for cognitive and motor assessments for children aged above 6 years on which to compare to this new touch screen assessment tool, so other approaches could be used that utilize three-dimensional local stimuli (Zuilkowski et al., 2016).

Secondly, the item battery should be expanded to include other domains, in particular, spoken and written language skills. Receptive and expressive language skills are vital for scholastic development and are key in the identification of learning difficulties, as language difficulties are widely associated with increased risk of poor educational outcomes (Tomblin, 2008; Peterson et al., 2009). For example, vocabulary knowledge is closely related to children's mathematics skills (Lee et al., 2004), and language proficiency is closely associated with academic attainment in the UK (Whiteside et al., 2016), and in low-income countries (Pretorius and Mampuru, 2007). Similarly, written language skills, especially literacy, are considered key building blocks on which later learning is dependent (e.g., Cunningham and Stanovich, 1997; Duncan et al., 2007; Sparks et al., 2014), so inclusion of tasks assessing spoken and written language processing would enhance the scope of this new cross-cultural touch screen assessment tool.

Finally, standardization is needed with the collection of normalized data across a large sample of pupils in low-to-high-income countries. This would afford comparisons between children's actual performance on the assessment tasks and their expected levels of performance based on developmental trajectories. This would aid in the identification of children in need of additional educational support and would enable the underlying nature of poor scholastic attainment to be investigated by profiling relative strengths and weakness in performance. For teachers to optimize the potential of this new cross-cultural assessment tool, guidance is required as to the interpretation of test performance and in how to scaffold individual learners identified at risk of underachievement and in need of intervention support.

## Conclusion

The attainment of a child's full development capability is considered a human right by the United Nations (Convention on the Rights of the Child, 1989, article 6^6^) and the early identification of children with disability is a high priority (World Health Organization, 2012^7^). For the first time, we have demonstrated that touch screen tablet technology can address this concern by providing a reliable and valid method of assessing core cognitive and motor skills, known to be associated with scholastic progression, in the early primary school years. The advent of touch screen assessment tools to evaluate early child development, such as the one described here, is important as this new technology will enable strengths and weaknesses of individual children to be determined, which will inform educators of those children most at risk of learning difficulties. This, in turn, will help to target educational interventions to those most in need, assuring no child is left behind. In addition, our touch screen assessment tool has been shown to be applicable across low- and high-income countries so it can be used to make cross-cultural comparisons of early child development. This will enhance theoretical understanding of generic factors and culturally-specific factors that are required for progress through school, especially for children at risk of learning difficulties, and will enable educational interventions to be evaluated at a global scale.

## Author contributions

NP designed the new touch screen tablet-based assessment and conducted Study 1. LO conducted Study 2 and analyzed both datasets. Both authors co-wrote the manuscript.

## Funding

The manuscript was prepared, in part, during the award of an ESRC-funded PhD Studentship (ES/J500100/1) to LO.

### Conflict of interest statement

The authors declare that the research was conducted in the absence of any commercial or financial relationships that could be construed as a potential conflict of interest.

## Footnotes

^1^The World Bank, “GDP per capita (current US$)” (The World Bank Group, 2015) http://data.worldbank.org/indicator/NY.GDP.PCAP.CD.

^2^Pearson Education Limited (2013). *Introducing Q-interactive*.Retrieved June 28, 2016 from http://www.helloq.co.uk/content/dam/ped/ani/uk/helloq/downloads/introducing-q-interactive.pdf.

^3^In Malawi children can repeat years if they fail to progress so our sample includes some older children who were repeating years 1–3 of primary school.

^4^Test-retest reliability for Mathematics was conducted with a third of the Malawi sample only, all of who received standard teaching practice across the 8-week interval between first and second administration. The rest of the Malawi sample received a specific mathematics intervention during the intervening 8-week period between assessments, which might have influenced test-retest reliability results, so these children were not included in the test-retest reliability measure for Mathematics.

^5^*Children and Parents: Media Use and Attitudes Report*. Ofcom Report (2014). Retrieved May 23, 2016 from http://stakeholders.ofcom.org.uk/binaries/research/media-literacy/media-use-attitudes-14/Childrens_2014_Report.pdf.

^6^United Nations (1989). *Convention of the Rights of the Child*. Retrieved March 5, 2015 from https://treaties.un.org/Pages/ViewDetails.aspx?mtdsg_no=IV-11andchapter=4andlang=en.

^7^World Health Organization (2012). *World Report on Disability*. Retrieved May 23, 2016 from http://www.who.int/disabilities/world_report/2011/report.pdf.
